# Functional Group and Substructure Searching as a Tool in Metabolomics

**DOI:** 10.1371/journal.pone.0001537

**Published:** 2008-02-06

**Authors:** Masaaki Kotera, Andrew G. McDonald, Sinéad Boyce, Keith F. Tipton

**Affiliations:** School of Biochemistry and Immunology, Trinity College, Dublin, Ireland; University of Michigan, United States of America

## Abstract

**Background:**

A direct link between the names and structures of compounds and the functional groups contained within them is important, not only because biochemists frequently rely on literature that uses a free-text format to describe functional groups, but also because metabolic models depend upon the connections between enzymes and substrates being known and appropriately stored in databases.

**Methodology:**

We have developed a database named “Biochemical Substructure Search Catalogue” (BiSSCat), which contains 489 functional groups, >200,000 compounds and >1,000,000 different computationally constructed substructures, to allow identification of chemical compounds of biological interest.

**Conclusions:**

This database and its associated web-based search program (http://bisscat.org/) can be used to find compounds containing selected combinations of substructures and functional groups. It can be used to determine possible additional substrates for known enzymes and for putative enzymes found in genome projects. Its applications to enzyme inhibitor design are also discussed.

## Introduction

Nomenclature is of fundamental importance in science [1]–[4]. Without reliable nomenclature systems, it would be difficult to know if each person in a discussion was talking about the same thing, and carrying out a literature survey would be almost impossible. Nomenclature not only gives a name to objects, but can also be used to characterize objects. Previously, nomenclature systems were the only way to look up chemical compounds or enzymes of interest. However, the rapid increase in the volume of literature and scientific data is making the use of computer algorithms unavoidable in the search for relevant data.

Missing connections between metabolites is a major problem of metabolic modelling. Just as gene-sequence studies have revealed many putative enzymes with unknown substrates (orphan enzymes), metabolomic studies are revealing a plethora of orphan substrates, which makes the need for rational approaches to identifying the enzymes involved in their formation and breakdown a pressing concern. In this context, orphan substrates may be defined in different ways. Poolman et al. [5] defined “orphan metabolites” as “metabolites involved in only one reaction” and “dead-end metabolites” as “metabolites involved in more than one reaction, but having no producing or no consuming reaction”. Although some metabolites, such as lactate, may be metabolic end products, in other cases the situation simply reflects a lack of knowledge. Both kinds of metabolite may cause the network to be unbalanced. Here we define an orphan substrate as one that is known to occur physiologically but neither the reaction to synthesize it nor degrade it are yet known. This kind of metabolite is problematic in metabolic-modelling studies, making it important to determine the possible reaction(s) in which it is involved. The same may apply to xenobiotics, many of which are either metabolized in some organisms or interact specifically with enzymes or “receptors”. A systematic approach based on the chemical structure of the metabolite should be of value in this respect.

The relationship between a chemical structure and its reactivity has been well investigated in pharmacology, the first step of which is pharmacophore searching prior to more detailed molecular analysis [see, e.g., 6–15]. There are a variety of tools for substructure searching, but their main purpose is drug design rather than novel pathway discovery. It is also hoped that BiSSCat will be useful for preliminary screening prior to more detailed molecular modelling studies and QSAR analysis.

In the field of organic chemistry, functional groups have been defined as atoms or atom groups that show relatively constant characteristics even when connected to different structures [3]. Researchers who are interested in chemical compounds in living organisms face several specific problems. They might want to find the common features of a group of substrates for certain enzymes, or how a group of substrates is converted into other types of compounds, even when the total structures are not specified. Interactions between proteins and small chemical compounds, including enzymatic reactions, follow the same rules that apply in organic chemistry but also have some specific characteristics. Recognition of small compounds and catalytic mechanisms is usually much more complex than that found with catalysts in organic chemistry, making it difficult to predict the fate of chemical compounds in living organisms.

The most reliable clue for guessing the function of putative genes is protein sequence similarity to well-investigated gene products, but such annotations have to be interpreted with caution. This is because they inevitably include uncertainty associated with each of the steps from enzyme studies to genome annotation. Most enzyme-specificity studies are not exhaustive, because experimentalists are generally interested in identifying the presumed physiological substrate(s) and inhibitor(s), or artificial substrates that make enzyme assays easier to perform. Substitution of even a single amino-acid residue can cause changes in terms of substrate specificity or reactivity. The label of being “similar to” well-investigated genes provides a suggestion about function, but does not necessarily describe functional identity, which further increases the uncertainty associated with annotations.

Although some enzymes have very narrow substrate specificity, others are known to display wider substrate specificity. Metabolome analyses have uncovered many secondary metabolites that appear to be species specific and it has been suggested that broad substrate specificity may contribute to metabolome diversity [16]. It has also been suggested that relaxed substrate and reaction specificities can have an important role in enzyme evolution [17]. Ideally, each of these enzyme specificities should be confirmed experimentally, however, it is practically impossible to check all enzymes for all compounds at present, as such experiments would be both costly and time-consuming to perform.

We propose that studies on enzymes or compounds that have been less thoroughly investigated should be made without making any assumptions about enzyme specificity. This provides a starting point for the consideration of possible combinations of recognized and putative enzymes (gene products) and their functions (enzyme reactions) in an expanding set of gene products and metabolites. Substrate specificity is generally described using a free-text description of the functional groups involved, the generic names of compounds, or one or more equations that describe the reaction(s) catalysed. These are subsequently used in genome annotations. Enzymes and their substrates are sometimes identified by class names. For example, the names alcohol dehydrogenase (EC 1.1.1.1) and amine oxidase (EC 1.4.3.4) give no indication of the breadth of the specificities of these enzymes. Indeed, it is likely that several possible substrates for such enzymes are not registered as substrates in reaction databases, because they have not been studied. Such a lack of precision highlights the need to make the relationship among compounds' names, class names, substructures and functional groups clear.

In this paper, we have defined substructures that include known functional groups, and made it possible to obtain chemical compounds from biochemical databases. We have also provided a web-based tool (http://bisscat.org/) for searching defined substructures and obtaining a list of compounds containing them. One can combine a number of defined substructures to produce more complicated substructures, and can search for enzymes based on functional groups. As an example of what can be achieved using BiSSCat, we have determined which substructures are commonly used by a particular group of enzymes, and then proposed some possible candidate compounds that could act as substrates of those enzymes. Since substructure and location are important for all ligand-binding processes, this approach should also be of wider value. Furthermore, it should help to connect nomenclature and machine-readable expressions of chemical compounds, and to fill in the gaps in our knowledge of genomic and metabolomic relationships.

## Results

The two major original parts of the BiSSCat dataset are SUBSTRUCTURE and FGROUP. The SUBSTRUCTURE part is constructed computationally and stores a collection of biochemical substructures. These were calculated using several different concepts, including the distinction between elements based on their electrostatic and physicochemical properties (Table 1). The FGROUP part comprises an index of names for functional groups and other biochemical substructures, which enables one to look up the substructure easily.

**Table 1:** Physicochemical properties defined in SUBSTRUCTURE.

Type of property	Content and abbreviations
Orbital	sp, sp2, and sp3 (sp, sp2, and sp3, respectively)
Number of attached non-hydrogen atoms	0, 1, 2, 3, and 4 (×0, ×1, ×2, ×3, and ×4, respectively)
Ring	Part of 3-, 4-, 5-, and 6-membered ring (r3, r4, r5, and r6, respectively)
Delocalized electrons and mobile hydrogens	Part of a conjugate bond (conj), a resonance bond (res), an aromatic ring (ar), an aromatic 5-membered ring (ar5), and an aromatic 6-membered ring (ar6)
Miscellaneous properties	electrically negative atoms (neg), nitrogen atom of an amide (namide), and carbon atom of a carboxylate group (cx).
Electrostatic properties	cation (ep1), anion (ep2), donor of a hydrogen bond (ep3), acceptor of a hydrogen bond (ep4), polar, which can be both donor or acceptor of a hydrogen bond (ep5), hydrophobic (ep6), and undefined properties (ep7).

The names used in FGROUP were assigned manually with the aid of the web-based BiSSCat substructure-search tool (described below). The merit of having this sub-database is that one can search for any substructure using a number of names without bothering about the definition of SUBSTRUCTURE entries unless one has a very complicated query. Most functional groups referred to in the IUBMB Enzyme List are covered, so the selection of FGROUP entries is currently biased for use with enzymatic reactions. For instance, many organic functional groups such as alcohols are further divided into their subgroups (primary, secondary and tertiary alcohols), whereas inorganic functional groups are not so detailed. It is hoped that BiSSCat users will give us feedback on any omissions. The database is designed so that a group of substructures can share one FGROUP, and a single substructure can belong to two or more FGROUPs. This rule might seem complicated, but it reflects the situation found in nature. For example, aldehyde, carboxylate, and amide groups belong to the carbonyl functional group, whereas the N-formyl group belongs to both the aldehyde and amide functional groups. Enzymes and other proteins often recognize more of a substructure than just the functional group(s), and the threshold for distinguishing between these is not always obvious. Therefore, FGROUP assigns names not only for functional groups but also for some larger substructures, such as sugars, which are specifically recognized by glycosyltransferases, glycosidases, *etc.*

The database currently comprises 241,709 chemical compounds whose non-hydrogen atoms are classified into 2,736 different ATOM entries. Each ATOM entry is given an ID number (ATOM0001–ATOM2736) based solely on its order of inclusion in the BiSSCat database. There are also 1,857,839 SUBSTRUCTURE entries in the database. Serial ID numbers are also assigned to these SUBSTRUCTURE entries (S0000001–S1857839) and, as discussed below, the IDs bear no relation to substructure type.

**Figure 1:**
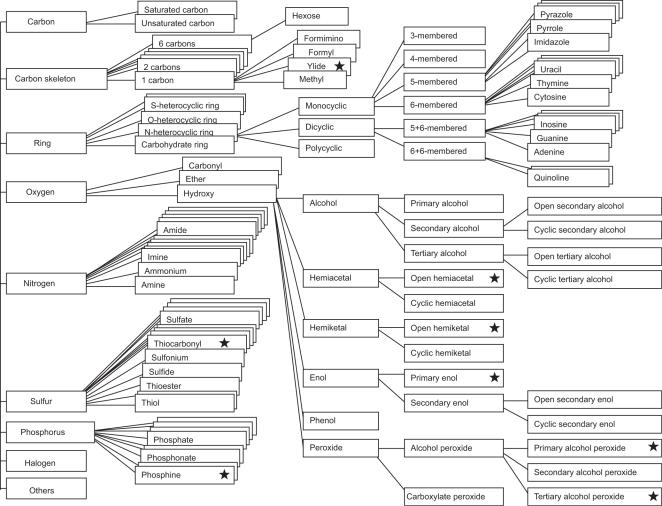
Partial classification tree of FGROUP: stars indicate FGROUP entries on which no enzymes are known to act. A complete list of FGROUP entries can be seen at http://bisscat.org/fgroup.html.

489 FGROUP entries were assigned for the current release (as of January 1, 2007). These correspond to 660,946 recognized SUBSTRUCTURE entries and to 4,964,487 non-hydrogen-atom locations in the KEGG [18] and NCI [19], [20] databases, which have been constructed for different purposes (containing mostly endogeneous compounds and xenobiotics, respectively) and have minimum overlap between them. An overall view of the classification of FGROUP entries is summarized in Figure 1 (the complete set defined to date is available at http://bisscat.org/fgroup.html). ID numbers are given to FGROUP entries in such a way that they approximate to a hierarchical classification. The FGROUP list does not strictly reflect classification of physicochemical or biochemical characteristics. Since the classification of some functional groups can be based on a number of different aspects, it is impossible to describe the classification of functional groups in a simple tree structure. There are 2,357 instances in the database where all atoms in a functional group are part of those in another functional group, and 8,625 cases where two functional groups share some atoms. The FGROUP list can be expanded to accommodate newly defined functional groups or substructures in the future.

Table 2 provides details of the numbers of SUBSTRUCTURE and FGROUP entries that occur in KEGG but not in the NCI databases and vice versa. This table also gives details of the number of substructures known to be involved in reaction centres and in enzyme reactions. Some SUBSTRUCTURE entries were found in KEGG that were not present in the NCI databases. These SUBSTRUCTURE entries contained “R” (representing omission of substructures such as alkyl groups) and “*” (representing repetition in polymers such as glycans, nucleotides and proteins). FGROUP entries found only in the NCI databases are functional groups for which no enzyme reactions have been recorded, such as ammonium ylide, thioaldehyde, phosphine, silane and stannane. The FGROUP entries in KEGG that are not in the NCI database include thiamine, fluorophosphate, chlorophyll, heme and cobalamin. These are listed on the BiSSCat website. Acyl halides and alkyl magnesium halides are important agents in organic chemistry, but were not found in either database because they are generally unstable under physiological conditions.

**Table 2:** Statistics on SUBSTRUCTURE and FGROUP entries in BiSSCat.

	Total	Shared	Unique in KEGG	Unique in NCI	Enzyme reactions	Reaction centres
ATOM	2,731	634	190	1,907	526	218
VICI	635,541	20,038	38,432	577,071	19,176	9,526
BOND	401,216	21,849	28,548	350,819	16,331	6,621
CONJ	188,280	735	6,632	180,913	2,626	1,894
FRAG	183,731	4,344	9,378	170,009	4,963	2,452
RING	384,578	1,722	28,510	354,346	8,368	3,742
SKEL	194,761	1,867	9,306	183,588	4,359	2,504
FGROUP	489	407	24	58	338	315

### The web-based substructure-search tool

**Figure 2:**
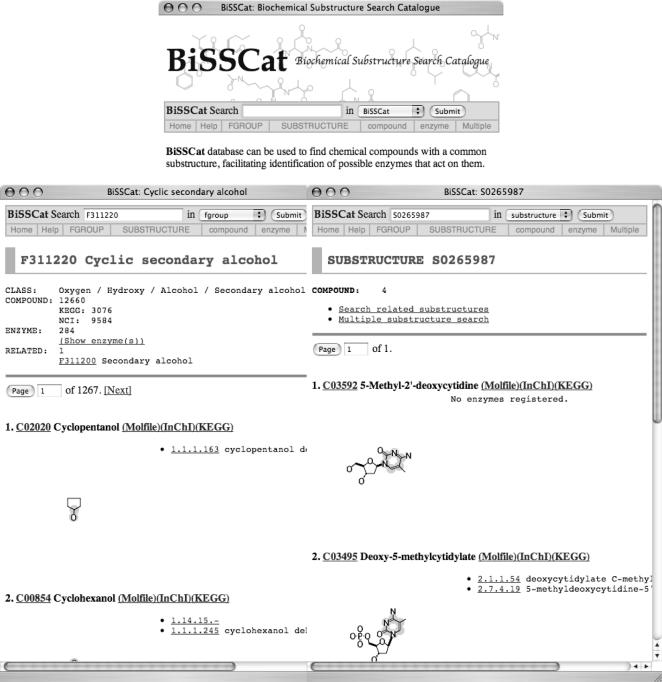
Screenshots of BiSSCat website: homepage (top), an example entry of FGROUP (left) and of SUBSTRUCTURE (right).

BiSSCat provides a number of alternative ways of looking up chemical compounds or biochemical substructures. Here we give an outline of the web-based program (http://bisscat.org/), and further details are provided on the website's help page. The user must install an Adobe SVG plug-in (http://www.adobe.com/svg/) and enable cookies in order to use these tools. Screenshots of the webpage are shown in Figure 2. Each chemical compound entry has an automatically generated interactive SVG image, which can be used to find the substructure of interest. The text-search option (located at the top of the homepage) can be used to search for (1) any term for compounds, functional groups, substructures and enzymes, (2) molecular formulae of compounds, functional groups and substructures and (3) EC numbers and other IDs registered in BiSSCat. One can use the text-search option to search the whole of BiSSCat or one can limit the search to compound, FGROUP, enzyme, reaction or SUBSTRUCTURE by selecting the item of interest from the drop-down menu.

Three types of objects, FGROUP (or SUBSTRUCTURE), compound and enzyme, can each be searched in three different ways (by name, tree and structure). The first way is using the alphabetically ordered list of these objects' names. The second way is to use the hierarchical classification tree. The difference between an FGROUP and a compound's classification can be explained using “hexopyranose” as an example. Hexopyranose is a word used to describe a class of compounds with the molecular formula C_6_H_12_O_6_, and containing four hydroxy groups and one cyclic hemiacetal within a six-membered ring. 22 hexopyranose compounds can be found in the current release of the database, with examples being glucose and mannose. The corresponding FGROUP entry shows the substructures involved in compounds such as glycans, of which, 1,661 compounds can be found.

The third way of searching the database, i.e. the structure search option, needs further explanation. Searches of FGROUP, SUBSTRUCTURE and compound entries can be based on elements, electrostatic and physicochemical properties, and graph topology. For example, aryl carboxylate contains C_2_O_2_ with the central carbon atom being a carboxylate carbon (cx), the other being an aromatic carbon (ar) and two oxygen anions (ep2). There are 17,925 SUBSTRUCTURE entries containing C_2_O_2_, which includes many FGROUP entries that are not carboxylates (e.g., F33400 Carboxylate ester). Among them, 2,988 entries have one carboxylate carbon and two negative oxygen atoms and these belong to six FGROUP entries that have “carboxylate” in their name (F331000 Carboxylate, F331100 Alkyl carboxylate, F331200 Allyl carboxylate, F331400 Aryl carboxylate, F331300 Acetylene carboxylate and F331400 2-Oxo carboxylate). 305 SUBSTRUCTURE entries are obtained when “aromatic” is added to the search condition.

Another option is to search for compounds based on structural information. Using the “Multiple Substructure Search” option, one can find compounds based on the presence or absence of substructures or functional groups. This can greatly increase the specificity of the search, and reduce the number of compounds to consider. For example, there are 55 compounds in the database that have “carboxylate” in their name but there are 22,160 compounds that contain the “carboxylate” structure. There are 685 compounds containing adenine in the database but there are only 62 compounds that contain both carboxylate and adenine. Of these, 28 of the compounds do not contain a thioester group.

FGROUP entries in reaction equations can also be searched to find enzymes. For example, reaction equations that include generic names such as “alcohol+NAD = aldehyde+NADH” can be searched. Partial equations, such as “alcohol = aldehyde” or “amine = aldehyde” can also be used.

### Enzyme reactions and substructures

There are currently more than 4030 enzymes with assigned EC numbers (see http://www.enzyme-database.org/) [21]. Enzyme classification is based on the reaction catalysed but the type of reaction given for different enzymes falls into different categories, as follows: (1) one specific reaction, (2) a reaction is given where one of the physiological substrates is not known, (3) a general reaction is assigned to represent large polymers such as a glycan, nucleic acid or protein, (4) a general reaction is given, because the enzyme has a wide substrate specificity, (5) two or more reactions are provided, where the enzyme catalyses the same type of reaction but with different substrates, and (6) a multi-step reaction is catalysed and the overall reaction is given a single EC number. In cases where an enzyme catalyses two or more distinctly different types of reactions, EC numbers are given to each of these reactions. The descriptors of reactant structures (Molfiles [22]) are incomplete in cases (2), (3) and (4), so it is difficult to automatically obtain the corresponding compounds from existing databases.

FGROUP entries can be used to obtain specific compounds in the case of (4) above, where a class name is used in the reaction equation. As an example, EC 1.1.1.1 (alcohol dehydrogenase) comprises enzymes that oxidize alcohols with the concomitant reduction of NAD^+^. The term “alcohol” does not refer to a specific chemical compound, but is a generic term used for any chemical compound containing one or more hydroxy groups. Suppose that we would like to identify a dehydrogenase that acts on a newly identified alcohol. EC 1.1.1.1 acts on a limited set of alcohols, although the substrate specificity of such an enzyme depends upon its origins, i.e., species and tissue. Different enzymes accept different sets of alcohols, but it is not known how substrate specificity could change in orthologous enzymes. The substrate specificity of this enzyme is clarified in the comments' section of the IUBMB enzyme entry, where it states that it “Acts on primary or secondary alcohols or hemi-acetals; the animal, but not the yeast, enzyme acts also on cyclic secondary alcohols”. The terms “primary alcohol”, “secondary alcohol”, “hemi-acetal” and “cyclic secondary alcohol” are registered in the BiSSCat database and there are 13,371, 20,230, 775 and 12,660 examples of each, respectively. In this way, FGROUPs can provide a number of possible substrates for enzymes described in generic expressions.

In the case of (5), as more than one specific compound is named as a substrate/product, it is possible to deduce substructures that are common to each substrate and/or product. For example, EC 2.1.1.50 (loganate O-methyltransferase) acts on two compounds, loganate and secologanate. The structural difference between these two substrates is, therefore, not sufficient to prevent recognition by the enzyme. Substructures were divided into two groups: those containing reaction centre atoms and those containing other substructures that might be recognized by the enzyme. A compound that has both of these attributes may be considered to be a possible candidate substrate for that enzyme.

In a preliminary analysis, candidate substrates were defined as those compounds having one substructure involving a reaction centre and at least three substructures found in a reported substrate for a given enzyme. Application of these criteria to the compounds in the BiSSCat database showed that 1,912 known substrates have more than 10 related structures that were, therefore, candidate substrates, 1,166 known substrates had between 1 and 10 other candidate substrates, and 934 had no alternative candidate substrates.

In cases where only a single specific reaction is provided, it is not possible to determine commonly used substructures, as there is no means of making a comparison. Some of the enzymes in the IUBMB Enzyme List appear to have narrow substrate specificities, so there might seem to be little need to predict other possible substrates. However, this may be a reflection of lack of knowledge. Furthermore, such information would be valuable if one needs to find the function of the corresponding orthologous gene products. Reaction centres can be defined as in the RPAIR database [23], [24]. The reaction equation itself is not enough to determine which substructures are recognized by an enzyme, although the BRENDA database (http://www.brenda.uni-koeln.de/) provides additional data on the specificities of many enzymes. In a situation where no information other than the reaction equation is available, the best one can do is to find compounds with the same types of atoms or functional group(s). Substructure searches of the BiSSCat database can be used to find atoms in the same environment. Among the compounds that are not currently known to be associated with any enzyme reaction, 62,402 compounds have the same type of atoms as those involved in reported enzyme reactions, and 2,182 of these are from the KEGG database.

One example is a group of compounds that include the 5-methylcytidine residue SUBSTRUCTURE entry (S0265987), i.e., deoxy-5-methylcytidine (1), DNA 5-methylcytosine (2), 5-methyldeoxycytidine diphosphate (3) and 5-methyl-2′-deoxycytidine (4). Deoxy-5-methylcytidine can be balanced in metabolic modelling as it is known to be involved in two enzyme reactions (EC 2.1.1.54 and EC 2.7.4.19). DNA 5-methylcytosine and 5-methyldeoxycytidine diphosphate are involved in only one reaction each (EC 2.1.1.37 and EC 2.7.4.19, respectively), and are examples of ‘orphan metabolites’, as defined by Poolman et al. [5]. Such orphan metabolites cause problems in metabolic modelling. No enzymes have been reported that act on 5-methyl-2′-deoxycytidine (4) but this does not cause the same types of problems as for compounds (2) and (3). However, it is expected that some reactions would involve compound (4) if it is naturally occurring. Substructure comparisons indicate that candidate enzymes would include deoxycytidine deaminase [EC 3.5.4.14] and deoxycytidine kinase [EC 2.7.1.74]. Two of the four compounds are involved in reactions that are catalysed by methyltransferases [EC 2.1.1.54 and EC 2.1.1.37 for (1) and (2), respectively], making it likely that methyltransferases also act upon compounds (3) and (4) to produce deoxycytidine diphosphate and 2′-deoxycytidine, respectively. The fact that EC 2.7.4.29 acts on both (1) and (3) also lends support to the presumption that there could be other enzymes acting on both compounds.

## Discussion

Although the approach taken in this study cannot ensure that a compound is truly a substrate for a given enzyme, it should help to minimize the number of candidate enzymes and compounds for experimental investigation. Further analysis of substructure changes during a reaction using RPAIR revealed that there were sometimes no corresponding products for the proposed substrates. A solution to this problem might be the addition of potential products to compound databases, however, it would first be preferable to confirm the existence of the predicted substrates/products experimentally, to avoid the inclusion of misleading information.

The BiSSCat substructure searching method is applicable to finding possible substrates having binding groups as well as a reaction centre. The process could also be applicable to identifying compounds that are unlikely to be substrates or might be inhibitors of a given enzyme. For example, EC 1.4.3.4 (monoamine oxidase) acts on many compounds that contain a primary amine group. If these substrates also contain a carboxy group, this can prevent the compound from being bound to the enzyme. The presence of an alpha-methyl group will not prevent binding of the substrate to the enzyme, but it does block the conversion of the substrate into the product. If information about binding groups and blocking groups is already known, BiSSCat can be used as an aid to the design of inhibitors. Such data are, in many instances, not presented explicitly in extant databases.

It is intended to further enrich BiSSCat with data about interactions between proteins and small compounds from the existing literature that are not in the present source databases and to incorporate results of future experiments. Several newer techniques, such as text mining of the enzyme-assay literature [25] and high-performance systematic assays to determine substrate specificity [26], can be applied. Needless to say, it is important to have a large collection of positive data, but the same can be said about negative data, i.e., compounds that the enzyme does not act on. It is meaningful to take account of compounds acting on enzymes in vivo, but it is also valuable to collect data about synthetic compounds that have not been observed in vivo. Information about mutated enzymes would also be valuable for enzyme-engineering purposes. It is intended to incorporate relevant data from more sources, including ERGO-light (http://www.ergo-light.com/) and UMBBD [27], in future developments of BiSSCat.

Given that the objectives of searching complete chemical structures and substructures are usually different, the search methods used are closely related to how they are represented. The first step of our method is to divide a chemical compound into its inherent substructures, which is similar to the first step in obtaining a systematic nomenclature for chemical compounds, such as obtained using IUPAC rules, and a variety of linear representations of chemical compounds, such as WLN [28]–[30], ROSDAL [31], SMILES [32], [33], SLN [34] and InChI [35]. The steps thereafter are different. When searching complete structures, the inherent substructures have to be arranged according to predefined rules, as it is essential that each chemical structure has only a single representation. This is not necessary for substructure searches, where users can freely modify the search criteria according to their needs. Graph-oriented algorithms applying maximum common subgraph isomorphism [36]–[39] are better than fragment code or fingerprint methods [40]–[46] in terms of precision when searching for compounds in databases that are similar to the query structure, although they present difficulties in terms of computational time (the graph isomorphism problem is NP-hard) and in the interpretation of the derived subgraphs.

Our method takes advantage of a pre-computed and assigned set of substructures, making the search speed faster and interpretation easier. The manual assignment of FGROUP was the most time-consuming process in the construction of the BiSSCat database, but it was an important step as it provides a direct correspondence between the generic names described in the IUBMB Enzyme List and the concrete substructures found in chemical-compound databases. This should make it easier for computer algorithms to distinguish between generic names and specific names. More importantly, it also makes it easier to understand the meanings of substructures found in computational analysis, which could help our understanding of the structure-function relationships of ligand-binding processes, including enzymes.

Both the database and search program have scope for further development, for instance by allowing the user to define distances between substructures, input substructures using SMILES or SMART format, or use a structure-drawing tool. These aspects will be addressed in future releases. We believe that our method should be of value in gene-product identification and in increasing our understanding of previously unknown metabolic pathways or drug-selection processes.

## Materials and Methods

### Data sources

The SUBSTRUCTURE database was constructed using data on the structures of 10,046 and 247,617 chemical compounds derived from the KEGG [8] and NCI [9], [10] databases, respectively, in MDL Molfile format. For convenience, the original database IDs assigned to compounds have not been changed, so that they can be used to link to the corresponding data in the source databases. Information on reported activities, such as enzyme substrates and products, is also provided so that one can search and analyse compounds using these data. Most compounds from KEGG are known to be involved in metabolism in living organisms. Most compounds from NCI include other valuable information, such as *logP*, the octanol/water partition coefficient [47], [48] as well as anti-cancer and anti-HIV screening results.

In order for a reaction to be catalysed, a chemical compound has to contain the appropriate functional groups, also referred to as the reaction centre. The KEGG/RPAIR database describes which atom in a substrate corresponds to which atom in a product in each enzyme reaction. The RPAIR database also defines reaction centre atoms, which undergo significantly more changes than other atoms in the reactant-pair during a reaction. These reaction-centre atoms are utilized in this study.

### Calculation of SUBSTRUCTURE

Biochemical substructures are computationally defined using seven attributes: atom (ATOM), vicinity (VICI), bond (BOND), skeleton (SKEL), ring (RING), fragment (FRAG) and conjugate (CONJ). Every substructure is represented as a graph object, with non-hydrogen atoms and bonds described as nodes and edges, respectively. Each substructure is distinguished in terms of its elements (C for carbon, N for nitrogen, etc.), electrostatic and physicochemical properties, and topology. Detailed definitions of the substructure types are provided below.

ATOM entries are distinguished by their elements and by their electrostatic and physicochemical properties, which are calculated for each non-hydrogen atom of each compound. Hydrogen atoms are not assigned individual ATOM entries, but are included with their adjacent non-hydrogen atoms. Table 1 shows the list of electrostatic and physicochemical properties defined in ATOM and other substructure entries. Most of these properties are based on the programmable atom typer program, PATTY [49]. Ring properties are an exception and they are explained later in this section. Physicochemical properties are provided for each non-hydrogen atom rather than for the total structure of the chemical compound. For example, while ethanol (CH3CH2OH) is a hydrophilic molecule, using the PATTY method, the two carbon atoms of the ethyl group (CH3CH2-) and the oxygen atom of the hydroxy group (-OH) are assigned as being “hydrophobic” (ep6) and “polar” (ep5), respectively.

VICI entries are defined in terms of ATOM entries. Other substructures (BOND, SKEL, RING, FRAG and CONJ) are defined in terms of VICI entries. A VICI entry is defined as a central atom and the atoms attached to it. Many functional groups correspond to VICI entries, e.g., carbamate, *N*-acetyl, and phosphate. A BOND entry is defined as a central bond between a pair of atoms, such as an amide bond. A SKEL entry is defined as a carbon skeleton/backbone, and examples include alkyl and aryl groups.

A RING entry is defined as a cyclic substructure, containing 3-, 4-, 5- and 6-membered, or larger, rings. Some common examples are the phenyl, imidazole and pyrrole rings. Ring properties are also added to each ATOM entry if the atom is part of a 3-, 4-, 5- or 6-membered ring. These additional properties were added as 3- and 4-membered rings have especially strong ring strain, which gives rise to their specific reactivities (such as EC 3.3.2.3, epoxide hydrolase, which acts on epoxide). 5- and 6-membered rings are ubiquitous substructures, as found in many sugars etc., and many reactions are known to produce 5- and 6-membered rings. Larger cyclic substructures are not described in ATOM entries but are included in RING entries.

A FRAG entry is defined as a fragment obtained when all rotatable bonds are cut. A rotatable bond is defined in the following way: only a single bond (saturated bond) that is not included in any ring substructure can be rotated. Amide bonds are not rotatable, as they are known to have an energy barrier that prevents rotation. Two cases that remain to be incorporated are where steric hindrance prevents rotation, and where an enzymic reaction helps rotation (such as occurs with *cis-trans*-isomerases). A bond consisting of one hydrogen atom and one non-hydrogen atom is also excluded. Using this definition, many biologically important polycyclic structures, such as purines, pyrimidines, hemes or sterols, are obtained. Considering rotatable bonds should also be helpful in understanding the conformational changes that occur when a chemical compound is accepted by an enzyme. In pharmacology, an important step of drug design is determining the number of rotatable bonds of possible medicinal compounds [50].

Finally, a CONJ entry is defined as a conjugated double or triple bond, i.e., a substructure with delocalized electrons. Technically speaking, CONJ entries are defined as connected sub-graphs consisting only of bonds where each of the two atoms has at least one resonance (res), conjugated double or triple bond (conj) or aromatic ring (ar) property. It is known that the delocalization of electrons leads to unique physicochemical characteristics and reactivities. In fact, CONJ includes many important substructures, such as 2-oxo carboxylate and triphosphate, which are found widely in biochemistry, and carotenoids and pheophytins, which are also found in pigments.

Substructures may be derived from other substructure types, which is the reason that IDs bear no relation to the type of substructure. For example, a phenyl ring is derived not only from the definition of RING entries, but also of FRAG entries (and CONJ entries in most cases). When a phenyl ring is connected to a heteroatom, the ring will also have a SKEL entry.
